# Assessment of the stated policies of prominent food companies related to obesity and non-communicable disease (NCD) prevention in Thailand

**DOI:** 10.1186/s12992-019-0458-x

**Published:** 2019-02-14

**Authors:** Nisachol Cetthakrikul, Sirinya Phulkerd, Nongnuch Jaichuen, Gary Sacks, Viroj Tangcharoensathien

**Affiliations:** 1 International Health Policy Program, Nonthaburi, Thailand; 2 Institute for Population and Social Research, Mahidol University, Nakhon Pathom, Thailand; 3 Deakin University, Geelong, Australia

**Keywords:** NCDs, Obesity, Private sector, Food company, Policy

## Abstract

**Objective:**

To review the publicly available policies and commitments of selected food companies in Thailand relating to obesity and non-communicable diseases (NCDs) prevention, and to assess these stated policies and commitments against global recommendations.

**Methods:**

Nineteen food and beverage companies, including 13 packaged food, three non-alcoholic beverage, two food retailer, and one fast food company were selected, based on their market share by sector and food category. A review of publicly available policies and commitments related to four domains (product reformulation, food marketing, nutrition information and food accessibility) was carried out for each company. Content analysis of all data was conducted, including a comparison of policy content against global recommendations in each domain.

**Results:**

Eleven companies (58%) reported at least one policy or commitment across the four domains. The packaged food companies reported policies in all four domains while the beverage companies committed to implement policies in all except the accessibility domain. The food retailers and fast food company only had policies in the reformulation and nutrition information domains. Very few of the policies and commitments covered all of the recommended components in each domain, and most lacked sufficient specificity to allow detailed monitoring and evaluation.

**Conclusion:**

A small number of the most prominent food companies in Thailand have several nutrition-related policies in place. However, these policies do not sufficiently cover recommended areas for NCD and obesity prevention. Moreover, the extent to which policy statements translate to implementation has yet to be evaluated. Successful implementation of nutrition-related policies by the food industry in Thailand will likely require concrete, measurable indicators to guide both corporate policy making as well as public monitoring. The Thailand Government requires greater capacity to establish effective multi-sector platforms for NCD prevention, and to evaluate food companies’ policies and enforce compliance both with regulations and voluntary commitments.

## Background

Burdens of obesity and diet-related non-communicable diseases (NCDs) are affecting countries worldwide [1, 2], and increasingly developing countries [2], including Thailand [3]. While there has been a gradual decrease in the prevalence of under-nutrition across the Thai population, the extent of over-nutrition has increased. The prevalence of over-nutrition rose almost fivefold between 1991 and 2014 among Thai men (from 7.7% in 1991 to 33% in 2014) and almost tripled among Thai women (from 15.7% in 1991 to 42% in 2014) [3].

Components of food environments, such as food availability, affordability and promotion, play an important role in influencing eating behaviours of individuals and populations [4, 5]. Large food companies now have a dominating presence in food environments globally, including major growth in the Global South in recent years [6].

Over the past decade, food environments in Thailand have changed dramatically. Food and beverage industries grew rapidly, providing greater supply of and demand for unhealthy foods and beverages. As an example, sales of savoury snacks grew by 27.0% from 71,800 metric tons in 2011 to 91,200 metic tons in 2016, while the soft drinks market grew by 26.9% from 2.7 billion litres in 2010 to 3.4 billion litres in 2015 [7].

To respond to changing food environments, there are policies, strategies and recommendations at the global and national level. Key examples are the World Health Organization’s (WHO) recommendations on marketing of foods and non-alcoholic beverages to children [8]; the SHAKE technical package on salt reduction [9]; and the Global Strategy on Diet, Physical Activity and Health [10]. In Thailand, examples of key actions taken by the Thai Government are the Ministry of Public Health Notification on nutrition labelling of energy, sugar, fat, sodium and use of Guideline Daily Amounts (GDA); front-of-pack labelling in five key food categories since 2016; and the introduction of a tax on sugar-sweetened beverages in 2017 [11].

The food industry has been widely criticised for manufacturing and marketing unhealthy foods [12–14] and undermining public health efforts, through its financial and political power, pressure, interference and influence over political processes [14, 15]. However, while the food industry has undoubtedly contributed to the current unhealthy state of food environments, it is also widely acknowledged that there is potential for the industry to contribute to creating healthier food environments [10, 16, 17]. For example, companies can limit unhealthy ingredients such as saturated fats, trans-fatty acids, free sugars, and salt through product reformulation [10]. Consequently, the WHO Global NCDs Action Plan [15] calls for the support of the food industry in creating and monitoring healthier food systems. The importance of collaboration with the food industry was also highlighted in other global recommendations [8, 9], although the extent of recommended collaboration between food companies and governments is contested [18–21].

Previous studies have revealed that, internationally, several food companies have announced various voluntary commitments in the area of NCD prevention, such as commitments related to marketing to children [22, 23] and food reformulation [22]. Correspondingly, international non-governmental organisations and researchers have established monitoring systems to assess food company contributions to obesity and NCD prevention [24, 25], including through initiatives such as the Access to Nutrition Index (ATNI) [26] and INFORMAS (International Network for Food and Obesity/Non-Communicable Diseases Research, Monitoring and Action Support) [27]. These initiatives have revealed that while there are companies taking commendable action in some areas, on the whole, industry policies and commitments fall far short of best-practice recommendations [22, 27, 28].

In Thailand, there has been no systematic analysis of major food companies’ nutrition-related policies and commitments, or the extent to which these policies and commitments comply with global recommendations on obesity and NCD prevention. This study aimed to: (i) review the publicly available obesity and NCD prevention policies and commitments of selected food companies in Thailand, and (ii) assess these stated policies and commitments against global recommendations.

## Methods

The study protocol was adapted from the approach developed by INFORMAS for assessing private sector policies and commitments related to obesity and NCD prevention [24].

### Selection of the sample companies

For the purpose of this study, ‘private sector organisations’ were defined broadly to include companies producing packaged food (baked food; biscuits and snack bars; confectionery; ice cream and frozen desserts; ready meals; and sweet and savory snacks), non-alcoholic beverages, food retailers and fast food restaurants.

A two-stage process was applied for selection of the most prominent food companies in Thailand. Firstly, we compiled a shortlist of potential companies for inclusion using Thailand market share data in the 2015 Euromonitor Passport database. The aim was to cover the companies with the largest market share in each sector or food category.. Secondly, a Thai Expert Advisory Group (TEAG) was formed to select food and beverage companies from the shortlist, based on the expertise and experiences of the members of the TEAG. The TEAG consisted of two senior government officials, three university professors and three leaders of non-governmental organisations, who have direct experience in food and health-related policy making or delivery in Thailand, including monitoring private sector performance. Besides market share, company selection considered product types, company portfolios, and the availability of company policies and commitments in the public domain.

Eventually, 19 companies were selected: 13 packaged food companies, three non-alcoholic beverage companies, two food retailers, and one fast food company (Table 1).

**Table 1:** Characteristics of the 19 selected companies

ID code	Company	Food category/sector	Type^a^	Market share (% of total in its food category/sector)
*Packaged foods*				
PK1	President Bakery PCL	Baked food	Thai	28.5
PB1	JG Summit Holdings Inc	Biscuit and snack bar	Transnational	25.8
PB2	Ezaki Glico Co Ltd	Biscuit and snack bar	Transnational	13.6
PB3	Thai President Foods Public Co Ltd	Biscuit and snack bar	Thai	8.5
PC1	Mondelez International Inc	Confectionery	Transnational	27.3
PC2	Mars Inc	Confectionery	Transnational	9.8
PC3	Perfetti Van Melle Group	Confectionery	Transnational	4.7
PI1	Unilever Group	Ice cream and frozen dessert	Transnational	60.3
PI2	Nestlé	Ice cream and frozen dessert	Transnational	13.4
PR1	Charoen Pokphand Group	Ready meal	Thai	40.6
PS1	PepsiCo Inc.^b^	Sweet and savory snack	Transnational	24.1
PS2	BerliJucker Plc [BJC]	Sweet and savory snack	Thai	9.7
PS3	TaoKaeNoi Food & Marketing Co Ltd	Sweet and savory snack	Thai	8.2
*Non-alcoholic beverages*				
B1	CocaCola Co	Soft drink	Transnational	28
B2	Thai Beverage PCL	Soft drink	Thai	12.2
B3	Boon Rawd Brewery Co Ltd	Soft drink	Thai	9.1
*Fast food restaurants*				
F1	Yum! Brands Inc	Fast food restaurant	Transnational	13.3
*Food retailers*				
R1	Seven & I Holdings Co Ltd	Food retailer	Transnational	8.3
R2	Tesco Plc	Food retailer	Transnational	7

^a^ ‘Thai’ – a Thai-owned company that produces goods or services in Thailand and may also export them to other countries; ‘Transnational’ – a corporate company that is owned by a person or company from another country, which controls production of goods or services in two or more countries other than Thailand

^b^ PepsiCo is the owner of Lays Potato Chips, and other prominent brands of snacks

### Compilation of evidence on policies and commitments

The study collected each company’s policies and commitments relevant to four policy domains globally recognised as important to promote the healthy diets and prevent obesity and NCDs [15]. ‘Policy’ is defined as a company’s stated position, objective, or plan to manage an issue [22], while ‘commitment’ is what companies say that they will do [29]. The domains comprised of policies or commitments on product reformulation (including reductions of salt, saturated and/or trans fat, added sugar, and energy as well as improvement in overall product portfolio), food marketing to children (including the communication channels, settings and marketing techniques to be covered, and other marketing factors such as product, timing, viewing audience, placement and content of the marketing message), nutrition information (including labelling of the front and back of food packaging, health and nutrition claims, and provision of nutrition information either online or on menus, as relevant) and food accessibility (with a focus on availability and affordability of healthy foods).

Sources of data included company websites (both national and global), annual certified financial reports, Thai Government and university websites, and the largest newspaper database in Thailand (*Matichon Online*). Publicly available company policies and commitments, published between August 2011 and July 2016, were included in this study. An internet search (using Google’s search engine) was also conducted between September and October 2016 in order to locate additional relevant information, using the name of selected companies and policy areas as search terms.

### Data analysis

A content analysis was applied to describe the details of each policy using standardised analysis criteria, which were adjusted from INFORMAS [24] for each of the four policy domains. Although there are currently very few specific global recommendations in the area, company policies and commitments were compared with recommendations from relevant WHO reports [9, 8, 10, 15, 30] and previous studies in the area [22]. The findings were also compared with international good practice relevant to the Thai context, as compiled by INFORMAS (Table 2). In all cases, the focus was on company policies and commitments that went beyond simply complying with existing legislative requirements operating in Thailand.

**Table 2:** Global recommendations and examples of good practice on four policy domains relevant to the Thai context

Domain	Globally recommended actions	Selected good practice examples relevant to the Thai context
1. Food reformulation	Reducing the levels of saturated fats, trans-fatty acids, free sugars and salt in existing products [9, 10, 15, 30].	Unilever International and PepsiCo Global made commitments to reduce nutrients of concern including saturated fat, added sugars, salt, trans fat and energy content of certain products [52, 53].
	Reducing salt in foods and meals served at restaurants and catering outlets [30].	McDonald’s Australia committed to reformulating its burger buns to contain less sugar, reducing salt across cheese and chicken products, and frying in a vegetable oil blend [54].
2. Food marketing to children	Practising responsible marketing that supports the promotion of healthy diets and physical activity and the overall aims of the Global Strategy, particularly with regard to the promotion and marketing of foods high in saturated fats, trans-fatty acids, free sugars, or salt, especially to children [8, 10].	The International Food and Beverage Alliance fully supported the WHO Food Marketing Recommendations [55], which includes companies not directing any marketing communication for unhealthy products to children below 12 years of age [56].
3. Nutrition information	Providing consumers with adequate and understandable product and nutrition information, including simple, clear and consistent food labels (e.g. front and back-of pack nutrition labelling) and evidence-based nutrition and health claims [10, 44, 45]. This includes labelling the sodium content of foods and meals [57].	Nestlé Global made a global commitment across its product portfolio to only place a health or nutrition claim on a product when it complies with local regulations and the international Codex Alimentarius, and the claims must meet Nestlé’s nutrient profiling criteria [58]. It also committed all that its relevant food and beverage products worldwide will have GDA-based labels on front of pack [59]. PepsiCo Global committed to providing nutrition information on the side or back of its product packaging. Additionally, it will include nutrition information on nutrients for which a health or nutrition claim is made [60].
4. Food accessibility	Continuing to improve access to affordable, healthy and nutritious choices to consumers [10]	(Availability) Countdown New Zealand pledges to expand health and wellness sections in the supermarket and increase fruit and vegetable sales through active promotions. They also commit to having at least one confectionary-free checkout in every store [61]. (Affordability) Danone International committed to making healthier products at more affordable prices in low-income markets [62]. ALDI UK has offered discounted fruit and vegetables to customers since 2011 [63].

Company policies and commitments in each domain were compared across sectors and food categories, as well as by company type (transnational or Thai-owned). The study employed an independent review and analysis, with the overall interpretation being completed by three independent researchers. Two researchers independently reviewed and analysed the contents of policy statements and commitments related to the four domains. Discrepancies between the two researchers were discussed and resolved by a third researcher. Quotations from the documents, translated from Thai into English, are presented to illustrate certain particular issues.

## Results

Table 3 summarises the presence and absence of policies and commitments for each company by the four policy domains. Companies’ policies and commitments were retrieved from two sources – namely, their websites and the *Matichon Online* database. Across the four domains, 11 companies (58%), had stated at least one policy or commitment. Policies and commitment in relation to nutrition information where the most prevalent, with eight companies (42%) having policies in that domain.

**Table 3:** Policies and commitments of the selected companies

Policy areas and domains	Number of companies with available policies				
	Overall (%) (*n* = 19)	Packaged food (%) (*n* = 13)	Non-alcoholic beverages (%) (*n* = 3)	Fast food (%) (*n* = 1)	Food retail (%) (*n* = 2)
1. Product reformulation	7 (36.8%)	4 (30.8%)	1 (33.3%)	1 (100%)	1 (50%)
Salt reduction	3 (15.8%)	2 (15.4%) [PI1, PR1]	0	1 (100%) [F1]	0
Saturated/trans fat reduction	4 (21.1%)	3 (23.1%) [PI1, PR1, PS2]	0	1 (100%) [F1]	0
Added sugar reduction	3 (15.8%)	2 (15.4%) [PI1, PR1]	1 (33.3%) [B1]	0	0
Energy reduction	4 (21.1%)	2 (15%) [PI1, PS1]	1 (33.3%) [B1]	1 (100%) [F1]	0
Product portfolio improvements	3 (15.8%)	2 (15.4%) [PI1, PR1]	0	0	1 (50%) [R2]
2. Reduction of food marketing to children	3 (15.8%)	1 (7.7%)	2 (66.7%)	0	0
Restriction of all forms of media advertising to children	3 (15.8%)	1 (7.7%) [PI1]	2 (67%) [B1, B2]	0	0
3. Nutrition information	8 (42.1%)	5 (38.5%)	1 (33.3%)	1 (100%)	1 (50%)
Provision of back-of-pack nutrition information	3 (15.8%)	3 (23.1%) [PK1, PI2, PR1]	0	0	0
Provision of nutritional information either per serving or per portion	3 (15.9%)	1 (7.7%) [PI2]	1 (33.3%) [B1]	0	1 (50%) [R2]
Provision of front-of-pack nutrition information	5 (26.3%)	4 (30.8%) [PK1, PB3, PI1, PI2]	1 (33.3%) [B1]	0	0
Use of health and nutrition claims	1 (5.3%)	1 (7.7%) [PI1]	0	0	0
4. Food accessibility	3 (15.8%)	3 (23.1%)	0	0	0
Investment planned in improving accessibility	3 (15.8%)	3 (23.1%) [PK1, PB3, PR1]	0	0	0
Pricing ‘healthier’ products to be more consumers affordability	3 (15.8%)	3 (23.1%) [PK1, PB3, PR1]	0	0	0

### Product reformulation

There was at least one company that has developed a policy on reformulation in relation to one or more of its products. One of the packaged food companies has policies in relation to all five relevant areas of reformulation i.e. reductions of salt, saturated and/or trans fat, added sugar, and energy as well as improvement in its overall product portfolio. One of the soft drinks companies introduced policies to reduce sugar and energy in its beverage products. The fast food company stated that it aimed to reduce energy density in its menu items and all other nutrients (not including sugar). One of the food retailers stated that it intends to reduce salt, fat and energy. While most of the company commitments were non-specific in nature, without quantitative targets, several companies did specify relevant reduction targets or achievements as part of their policy/commitment statements.

*“We aim to reduce 15–20% salt in original products in order to accelerate reduction in salt intake among [the] Thai population to 5 grams per day by 2013. We formally announced our [nutrition-related] vision and goal.”* (UNILEVER GROUP)

“*We, at BJC, released a new product… where 100% rice bran oil is used in this product with 50% reduction in saturated fats.”* (BJC)

### Food marketing to children

The study found that three of the packaged food and beverage companies had introduced policies and commitments on reduction of marketing – such as advertising and direct sales – to children. These policies and commitments applied to marketing directed to children under 12 years old only. They explicitly applied to television and radio, in print and online media (including company-owned websites), as well as settings such as primary schools, but do not cover other marketing channels (e.g. food packaging). The policies and commitments specified that they applied to foods high in saturated fats, trans-fatty acids, free sugars, or salt, but did not provide further details of how this would be assessed.

*“Market responsibly, including no advertising to children under 12 anywhere in Thailand.”* (COCACOLA CO)

*“Direct sales to schools that focus on water, juice, milk and low-calorie beverages to support healthy nutrition habits among (school) children.”* (PEPSICO INC)

### Provision of nutritional information

The vast majority (eight companies) of company commitments in the area of nutritional labelling focused on compliance with government regulations regarding GDA nutrition labelling and Notification of the Ministry of Public Health (No. 367) B.E. 2557 (2014): Labeling of Prepackaged Foods. However, not all companies explicitly indicated that their policy is to comply with relevant labelling regulations.

Over and above commitments to comply with relevant regulations, some companies committed to provide additional nutritional information. Three companies (including two packaged food companies and a food retailer) stated that they provide nutrition information on per serving or per portion basis; although no companies committed to provide information per 100 g/100 mL. One company indicated that it provides clear nutrition and health claims on packages, but did not include additional details or relevant criteria for doing so. There were no other commitments regarding front-of-pack labelling. The fast food company committed to provide nutrition information on its menus or menu boards, and on its company websites, with no specification of the format for this information.

### Food accessibility

In this study, only three packaged food companies stated policies and commitments on availability and affordability of healthier products. They referred to developing company investment plans to improve delivery of and access to their healthy foods for children and rural populations. They also indicated policies to offer their healthier food products at more affordable prices. However, they did not define the level of affordable price, or what they meant by healthy products.

*“Creating safe, high quality, nutritious and affordable products is one of our key policies in increasing product availability for consumers... We have been working in partnership with the Rural Lives’ Development Foundation under the support from [a company] group and other partners on the ‘Raising Layer in Schools for Student’s Lunch Project’… [The project] supports students in rural areas to have access to quality foods.”* (SEVEN & I HOLDINGS CO LTD CHAROEN POKPHAND GROUP)

### The difference between Thai-owned and transnational companies

Across the sample of selected companies, a higher proportion of transnational companies had commitments to product reformulation than Thai-owned companies; but similar proportions were observed for marketing to children and nutrition information (Table 4).

**Table 4:** Comparison policies and commitments between Thai-owned and transnational companies

Domains of globally recommended actions	Number of selected companies with available policies and commitments		
	Overall (%) (*n* = 19)	Thai (%) (*n* = 7)	Transnational (%) (*n* = 12)
1. Food reformulation	7 (36.8%)	2 (28.6%) [PR1, PS2]	5 (41.7%) [PI1, PS1, B1, F1, R2]
2. Food marketing to children	3 (15.8%)	1 (14.3%) [B2]	2 (16.7%) [PI1, B1]
3. Nutrition information	8 (42.1%)	3 (42.9%) [PK1, PB3, PR1]	5 (41.7%) [PI1, PI2, B1, F1, R2]
4. Food accessibility	3 (15.8%)	3 (42.9%) [PK1, PB3, PR1]	0

## Discussion

This study analysed the publicly available policies and commitments of major food and beverage companies related to obesity and NCD prevention in Thailand. The study found that the majority of the selected companies adopted policies in at least one of the four policy domains recommended by WHO. However, the content of the policies varied across companies. Very few of the policies and commitments covered all of the recommended components in each domain, and most lacked sufficient specificity to allow detailed monitoring and evaluation. The transnational companies were found to have a greater number of stated commitments to implementing the global recommendations than the Thai companies, in particular on product reformulation. This may be due to global pressure on these transnational corporations to take concrete action to prevent obesity and NCDs.

Currently, there are no globally agreed (e.g. by the WHO) ‘gold standard’ instruments available to assess the specific contents of company policies in the four domains at the national level. However, there are several assessment tools developed by civil society groups, such as the ATNI [28] and the BIA-Obesity (Business Impact Assessment on Obesity and Population-level Nutrition) tool [27] developed by INFORMAS, that could be used to assess company policies and commitments at the national level, with appropriate tailoring of assessment measures to the country context.

In response to global recommendations [9, 10, 15, 30] for reducing the levels of saturated fats, trans-fatty acids, free sugars and salt in existing products, several companies had developed policies in these areas. Although WHO proposed a relative target of 30% reduction of daily salt intake [15], there are no international standards on levels of reduction of salt and other key nutrients (except trans fat) by food category. Furthermore, lack of specificity and consistency across companies makes it difficult to benchmark company actions in this area. In this context, governments need to develop their own targets for key nutrients and regularly monitor progress towards their national targets, similar to the UK Government actions on salt reduction [31, 32]. Strong regulation and close monitoring are effective methods for product reformulation, as shown by recent studies [33, 34] on salt reduction in ultra-processed foods. Mandatory product reformulation alone could achieve a reduction of salt content of 1.45 g/day.

Despite the existence of some company self-regulation of food marketing to children as recommended by WHO [8, 10], there is mounting evidence that, in its current form, self-regulation in this area is unlikely to be effective [35]. This is because current commitments in the areas have narrow definitions of many key components, such as the products included, the age to which policies apply (typically age 12, as compared with the definition of children as those under 18 in the United Nations Convention on the Rights of the Child [36]), the conceptualisation of ‘child-directed advertising (as compared with advertising to which children are exposed), and the narrow definition of marketing techniques and channels included (specific as opposed to general and all-encompassing). In addition, there is a lack of enforcement and effective sanctions for non-compliance. Despite some voluntary self-regulation of marketing to children in Thailand such as the Thai-pledge adopted in 2008; the frequency of unhealthy food advertisements remained high [37] where violations of the self-regulatory code are common [38, 39]. A recent study [40] reported 16.3 and 10% of sweetened beverages and savory snacks, respectively, had targeted advertisements to children in free and digital television programmes. Failure of self-regulation in reducing unhealthy food advertising to children has been reported in other countries; for instance, a study in Australia showed no reduction in children’s exposure to advertising of unhealthy fast food [41]. Thus, other models of regulation are likely to be needed, such as mandatory regulation or co-regulation, for which much greater government involvement would be needed in developing company commitments, ensuring strong compliance monitoring frameworks, and meaningful sanctions for non-compliance [42].

Nutrition labelling was the most common policy stated by Thai-owned and transnational companies. Compliance to nutrition labelling has increased as a result of a new regulatory requirement in 2016 by the Thailand Food and Drug Administration on mandatory GDA labelling in five categories of packaged foods: snack foods, chocolate, bakery, semi-processed foods and chilled and frozen ready-to-eat meals, as well as new requirements on nutrition fact labelling and health and nutrition content claims [43]. It is consistent with WHO and Codex Alimentarius that recommended food companies should provide adequate and understandable product and nutrition information on the food labels [10, 44, 45]. Despite the mandatory regulations in the area, there are variations in the content, scope and formats of nutrition labelling. Therefore, governments need to monitor regulatory compliance by food companies. In addition, and in acknowledgement of the lack of strong voluntary action from companies in this area, the Thai Government needs to explore additional nutrition labelling requirements, such as mandatory back-of-pack labelling per 100 g/mL, interpretative front-of-pack labelling (such as the Health Star Rating system from Australia or the NutriScore system in France [46]), and warning labels for products high in certain nutrients (as is the case in Chile [46]).

Thai companies showed some stated commitments to policies in promoting availability and affordability for healthier food choices, which is consistent with the Global Strategy on Diet, Physical Activity and Health [10]. In contrast, the transnational companies investigated in this study did not state these policies. The reasons for this difference between Thai and transnational companies are not clear, and beyond the scope of this analysis. Nevertheless, even where policies existed, commitments were vague and lacked clarity on key concepts such as healthier food choices, affordability, and target groups for implementation. There are several international examples of actions in this area that may serve as benchmarks of good practice, for example Nestlé have fortified some of their products in the Global South and established food access programmes in rural areas in Brazil [47, 48]. However, government tax policies and incentives may be necessary to drive this agenda. For instance, to increase the number of healthier food retailers in underserved areas, government could set incentives, tax policies and rules for retailers in rural areas or support the use of local food ingredients [49]. However, government will need to be aware of the impact on primary producers of lowering prices of healthy food.

Our findings on affordable healthier food choices are consistent with the 2018 Global ATNI report [29], illustrating that although the accessibility score increased from 0.9 in 2016 to 2.4 in 2018, it is the lowest scoring of all indicators. In other words, food companies are focusing less on affordability and accessibility of healthier products than other areas [50]. Similarly, most food companies in India do not consider the importance of providing access to affordable, healthier foods, particularly to low-income consumers, and do not appear to have developed any commitments or policies in this area [51].

The findings of this study are similar to those of studies in Australia, New Zealand and Fiji [22], particularly regarding the lack of publicly available policies and large variation in the policies and commitments across companies. However, some differences among the countries were observed, especially with regard to the policy content. Taking product reformulation as an example, some countries tended to focus on salt reduction and changes to the make-up of overall product portfolios while in Thailand, companies focused on addressing saturated fat and energy reduction.

This study has some limitations. Firstly, data collection relied only on publicly available information and thus did not cover internal policy documents and may therefore have missed information embedded in unpublished documents. In addition, for some of the transnational companies included in the study, policy information was sourced from the companies’ global websites, and the extent to which their policies related to the Thai context, or the degree to which they were applied in Thailand, was not clear. Secondly, the extent of policy implementation by these companies was outside the scope of this study, hence limiting knowledge of the extent to which policies are implemented as stated.

Future research needs to address gaps between policy and implementation, which can be useful for understanding company motivations and actions, holding them to account for their policies, and supporting improved government policy and action. Moreover, monitoring of food environments is needed for determining whether the available policies and commitments made are turned into actions. This evidence will assist the Thai Government in determining their policy responses to obesity and NCD prevention.

## Conclusion and policy implications

A small number of the most prominent food companies in Thailand have several relevant policies in place supporting obesity and NCD prevention. However, even for companies that have some policies in place, these do not cover all areas recognised as important, and the policies are not in line with global recommendations for their role in addressing NCDs.

For governments and the public health community, it would be beneficial to translate global NCD prevention recommendations into concrete measurable indicators that address the priority challenges with respect to creating healthier food environments. This will make it easier for companies to develop clear and meaningful policies, and will facilitate effective monitoring and benchmarking of performance. In addition, systems for monitoring the healthiness of food environments, as well as company policies and actions in the area need to be strengthened. In the absence of progress and greater industry action, governments need to consider stronger regulatory action and increased regulatory capacity.
